# Unravelling the challenge of cotrimoxazole and rifampin resistance in *B*. *melitensis* and *B*. *abortus*: A systematic review and meta-analysis

**DOI:** 10.1371/journal.pntd.0012630

**Published:** 2024-12-02

**Authors:** Masoumeh Beig, Elaheh Ebrahimi, Safoura Moradkasani, Forough Goodarzi, Mohammad Sholeh, Narges Golab

**Affiliations:** 1 Department of Bacteriology, Pasteur Institute of Iran, Tehran, Iran; 2 Department of Microbiology, School of Medicine, Tehran University of Medical Sciences, Tehran, Iran; 3 Department of Bacteriology, Faculty of Medical Sciences, Tarbiat Modares University, Tehran, Iran; 4 Student Research Committee, Pasteur Institute of Iran, Tehran, Iran; Mahidol Univ, Fac Trop Med, THAILAND

## Abstract

**Background:**

Brucellosis caused by *Brucella* (*B*. *abortus*) and *Brucella melitensis* (*B*. *melitensis*) poses a significant threat to human and animal populations. The World Health Organization (WHO) recommends rifampin and cotrimoxazole as first-line treatments for pediatric brucellosis. However, emerging resistance to these antibiotics raises concerns regarding their continued efficacy. This systematic review and meta-analysis aimed to quantitatively assess the prevalence of rifampin and cotrimoxazole resistance in *B*. *abortus* and *B*. *melitensis*.

**Methods:**

Eligible studies were identified by systematically searching various databases, such as Scopus, PubMed, Web of Science, and EMBASE databases, using specified search terms until 18 June 2024. The inclusion criteria required studies in English to report the resistance proportion with sample size details. The meta-analysis utilized a random-effects model to assess heterogeneity using the Q-test and I^2^ statistic. Meta-regression and subgroup analyses explored temporal, geographical, and guideline-related variations in resistance prevalence.

**Results:**

Among the 905 records, 59 studies spanning 21 countries (1976 to 2024) met the inclusion criteria. The prevalence of cotrimoxazole resistance, based on 3,756 isolates, was 0.034 (95% CI, 0.017, 0.068), with increasing trends over time, especially in *B*. *melitensis*. Rifampin resistance, involving 3,938 isolates, had a prevalence of 0.046 (95% CI, 0.027, 0.077), showing temporal and species-specific increases. Subgroup analyses revealed significant variations in resistance based on temporal, geographical, and guideline-related factors.

**Conclusions:**

This systematic review and meta-analysis highlighted an alarming rise in cotrimoxazole and rifampin resistance in *B*. *abortus* and *B*. *melitensis*, particularly in pediatric brucellosis. Temporal, geographical, and species-specific variations underscore the dynamic nature of antibiotic resistance, emphasizing the need for targeted interventions, surveillance, and global collaboration to preserve the efficacy of essential antibiotics in brucellosis treatment. The limitations include potential biases and the retrospective nature of the included studies, emphasizing the urgent need for standardized surveillance methodologies and robust reporting mechanisms.

## 1. Introduction

Brucellosis, a zoonotic disease caused by *Brucella* (*B*. *abortus*) and *Brucella melitensis* (*B*. *melitensis*), poses a significant threat to humans and animals [1–3]. Rifampin and cotrimoxazole are antibiotics commonly used to treat various infections, including those caused by *B*. *abortus* and *B*. *melitensis* [4]. These antibiotics are essential in pediatric cases because of their efficacy and safety profiles [5]. The World Health Organization (WHO) recommends using rifampin and cotrimoxazole as first-line treatment for children’s brucellosis [6,7]. However, recent studies have reported the emergence of rifampin and cotrimoxazole resistance in *Brucella* species, raising concerns regarding the effectiveness of these antibiotics in the pediatric population [8,9]. This resistance is particularly significant, as these antibiotics are often the first choice for treating brucellosis in children. Studies have documented rifampin- and cotrimoxazole-resistant *B*. *melitensis* isolates in China [10] and high rates of probable rifampin resistance among these isolates in Egypt [11]. In addition, cotrimoxazole and rifampin have been widely used in Africa to treat various infections, such as brucellosis, owing to their availability and cost-effectiveness [12–14]. However, the emergence of cotrimoxazole resistance in *Brucella* isolates, particularly in *B*. *melitensis*, poses a challenge to its continued efficacy in pediatric populations [12].

Similarly, rifampin resistance in *B*. *melitensis* isolates has been reported, indicating a potential decrease in the effectiveness of this antibiotic in treating brucellosis [15]. These findings underscore the growing concern regarding the efficacy of rifampin and cotrimoxazole in treating pediatric brucellosis. Additionally, these antibiotics exhibit a limited inhibitory effect against *Brucella* strains, meaning they are less effective at preventing the growth and proliferation of these bacteria. For instance, studies have shown that certain *Brucella* strains exhibit resistance or reduced sensitivity to these antibiotics, complicating effective disease management [11,16].

Considering the widespread prevalence of *B*. *melitensis* and *B*. *abortus*, the 2 most critical zoonotic agents of brucellosis [1], it is essential to consider alternative treatment options and antimicrobial combinations for pediatric brucellosis, especially in regions where rifampin and cotrimoxazole resistance is prevalent [17]. The susceptibility of *Brucella* isolates to other antimicrobial agents such as doxycycline, tetracycline, and fluoroquinolones should be carefully evaluated to ensure effective treatment in pediatric cases [10]. Furthermore, the potential use of novel antimicrobial agents and alternative treatment strategies, such as nano-sized particles, should be explored to address the challenge of antibiotic resistance in pediatric brucellosis [18]. The resistance of *B*. *melitensis* to rifampin was further substantiated by Brangsch and colleagues, who reported that *ropB* mutations account for a significant proportion of rifampin-resistant *Brucella* isolates [19]. Collectively, these findings emphasize the urgent need for comprehensive surveillance and management strategies to address the emerging resistance to rifampin and cotrimoxazole in pediatric brucellosis [20]. The emergence of rifampin and cotrimoxazole resistance in *B*. *abortus* and *B*. *melitensis* isolates has significant implications in treating pediatric brucellosis. Increasing reports of resistance to these pathogens pose a substantial challenge to effective treatment in children. Given the limited alternative treatment options and the potential severity of the disease, there is an urgent need for further research to understand the mechanisms of resistance and develop effective therapeutic strategies. Alternative treatment options and antimicrobial combinations should be carefully considered to ensure the continued efficacy and safety of brucellosis management in children. This study conducted a systematic review and meta-analysis to determine the prevalence of rifampin and cotrimoxazole resistance in *B*. *melitensis* and *B*. *abortus* isolates, considering the significance of these antibiotics in the recommended treatment by WHO.

## 2. Methods

### 2.1. Eligibility criteria

The inclusion criteria for articles in our systematic review and meta-analysis were studies that thoroughly examined and reported the resistance proportion, specified the sample size, and were published in full text in English. Only cross-sectional published articles studies were included. In addition, we did not exclude any patient populations in our analysis; all studies reporting rifampin or cotrimoxazole resistance prevalence were included, regardless of the type of population studied. The exclusion criteria applied to studies written in languages other than English, case reports, single-arm studies, cohort studies, pharmacokinetic studies, and studies with a sample size of fewer than 3 isolates.

### 2.2. Search strategy

Our systematic search was conducted using reputable online databases, including Scopus, PubMed, Web of Science, and EMBASE, until 18 June 2024. The search syntax is outlined as follows: (“*B*. *melitensis*” OR “*B*. *abortus*” OR “*B*. *abortus*" OR "*B*. *melitensis*" OR "brucellos*" OR "malta fever" OR "fever, malta" OR "gibraltar fever" OR "fever, gibraltar" OR "rock fever" OR "fever, rock" OR "*Brucella*" OR "cyprus fever" OR "fever, cyprus" OR "fever undulant" OR "undulant fever") and ("sensitivity tests, microbial" OR "antimicrobial susceptibility breakpoint determination" OR "test, microbial sensitivity" OR "tests, microbial sensitivity" OR "drug sensitivity assay, microbial" OR "microbial sensitivity test" OR "sensitivity test, microbial" OR "antibiogram*" OR "bacterial sensitivity tests" OR "tests, bacterial sensitivity" OR "sensitivity test, bacterial" OR "sensitivity tests, bacterial" OR "test, bacterial sensitivity" OR "bacterial sensitivity test" OR "antibiotic resistance" OR "drug resistance, microbial" OR "antimicrobial resistance" OR resistan* OR susceptib*) AND (cotrimoxazole OR TMP/SXT OR TMP-SXT OR Sulfamethoxazole-Trimethoprim OR rifampin OR rifampicin*), we adapted the search syntax according to each database’s guidelines. The protocol for conducting this meta-analysis was registered in PROSPERO with registration code CRD42023490423.

### 2.3. Selection process

Following the systematic online database search, all results were imported into EndNote (version 20), and duplicates were removed. To minimize bias, 2 authors (SMK and FG) independently searched and analyzed relevant publications. Disparities were resolved through a discussion with a third author (NG).

### 2.4. Data collection process

#### 2.4.1. Data items

The extracted data encompassed the first author, publication year, country, diagnostic method, sample source, number of positive tests, and total sample size. Two authors (MB and EE) independently extracted the data and resolved discrepancies through mutual agreements to ensure accuracy.

### 2.5. Study risk of bias assessment

Given the inclusion of cross-sectional studies, the Joanna Briggs Institute (JBI) checklist was used to assess the quality of the included articles [21]. Two authors independently performed the quality evaluation (MSH and SMK), and the third author addressed any discrepancies.

### 2.6. Synthesis methods

The number of resistant isolates and sample size were used to calculate the proportion of cotrimoxazole and rifampin resistance in *B*. *melitensis* and *B*. *abortus* strains, which was the first outcome of this study.

### 2.7. Statistics

The analysis utilized proportion as the outcome measure and employed a random-effects model. Heterogeneity, represented by τ^2^, was estimated using the DerSimonian–Laird estimator [22]. The Q-test for heterogeneity [23] and I^2^ statistics [24] have been reported. In the presence of heterogeneity (τ^2^ > 0), meta-regression analysis was conducted as a moderator for several years, and subgroup analysis explored prevalence differences between countries, continents, before and after 2020, strains, specimens, antimicrobial susceptibility testing (AST) methods, AST guidelines, and risk of bias.

To address the issue of zero events in some studies, we used the logit transformation method for our analysis. This approach is particularly effective in stabilizing variances and providing reliable estimates when dealing with proportions close to 0 or 1. We added 0.5% to studies with zero events to mitigate this issue. Our choice of the logit method over alternatives like the Freeman–Tukey transformation, which tends to overestimate resistance rates, ensures more accurate and reliable results.

Studentized residuals and Cook’s distances were employed to identify potential outliers and influential studies in the model [25]. Studies with studentized residuals larger than the 100 × (1 − 0.05 / (2 × k))th percentile of a standard normal distribution were considered potential outliers (using a Bonferroni correction with two-sided α = 0.05 for studies included in the meta-analysis). Studies with Cook’s distances more extensive than the median plus 6 times the interquartile range of Cook’s distances were considered influential. The funnel plot and Doi plot were used for the assessment of publication bias. Funnel plot asymmetry was assessed using the rank correlation and regression tests with the observed outcomes’ standard error as a predictor. The analysis used R (version 4.2.1) and the metafor package (version 3.8.1) [26–28].

## 3. Results

### 3.1. Descriptive statistics

**Fig 1:**
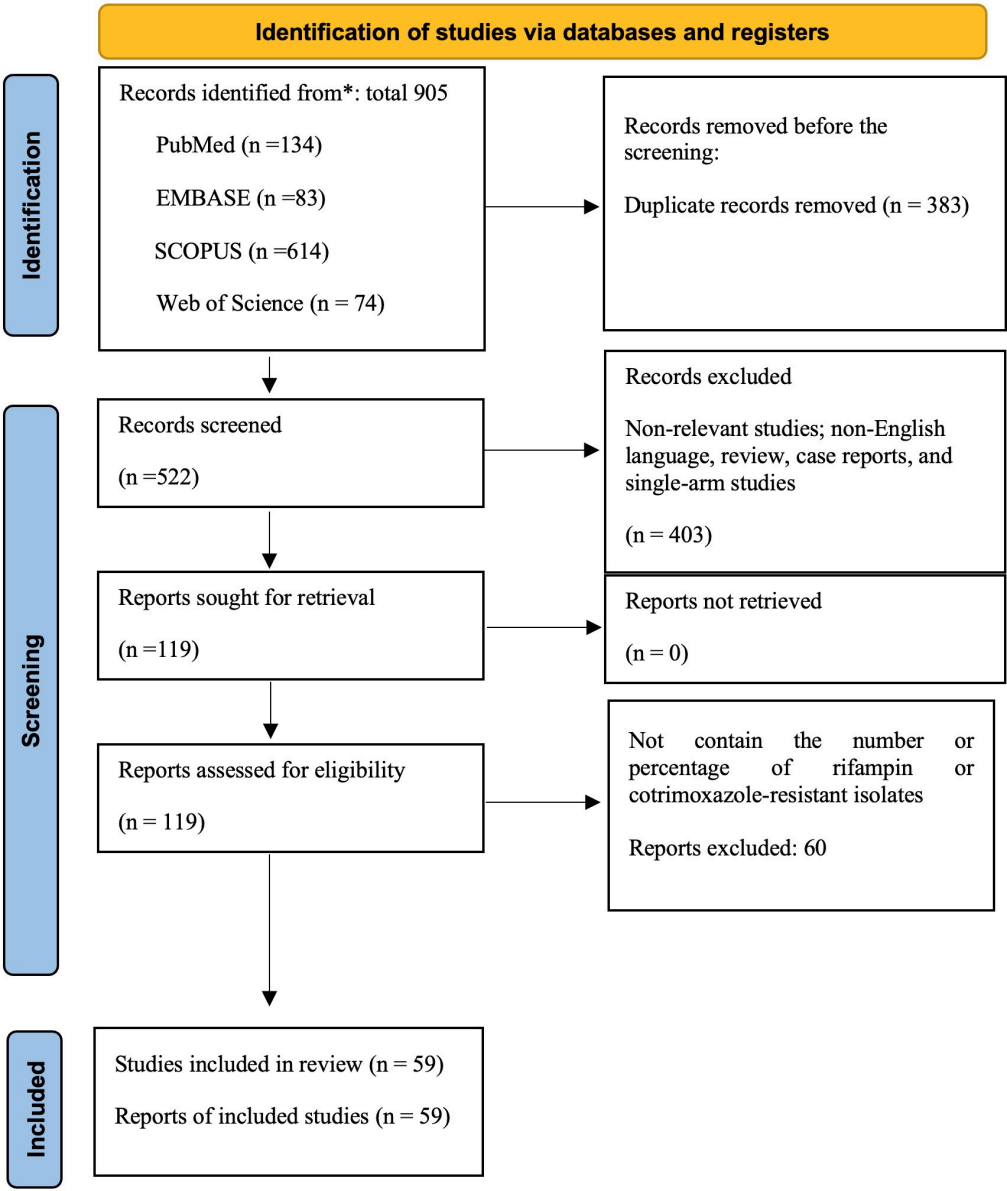
PRISMA flow diagram summarizing the article selection procedure.

The systematic online database search yielded 905 records. After eliminating 383 duplicates, 522 articles underwent initial screening. Subsequently, 119 articles were fully assessed, leading to the exclusion of 60 based on specific criteria. The final selection for this systematic review and meta-analysis consisted of 59 eligible studies [8,10,15,16,29–82]. A visual representation of the screening and selection process is presented in the PRISMA flowchart (Fig 1 and Table A in S1 File). The reports included in the study originated from 21 countries, encompassing 5 continents (United Kingdom, Iraq, Saudi Arabia, Spain, Turkey, Kuwait, Greece, Italy, Iran, China, United States, Peru, Trinidad and Tobago, Syria, Egypt, Kyrgyzstan, Malaysia, Brazil, Kazakhstan, Norway, Bosnia and Herzegovina), from 4 continent (Europe, Asia, Americas, Africa) spanning 1976 to 2024.

### 3.2. Comprehensive overview of antibiotic resistance prevalence

The proportion of cotrimoxazole resistance through 53 reports, with 265 resistant isolates among 3,756 investigated isolates, was 0.034 (0.017, 0.068), and heterogeneity between reports was significant (I^2^ = 90.32%, *p* < 0.001). The proportion of rifampin resistance through 60 reports, with 544 resistant isolates among 3,938 investigated isolates, was 0.046 (0.027, 0.077), and heterogeneity between reports was significant (I^2^ = 92.39%, *p* = 0.039).

#### 3.2.1. Prevalence of cotrimoxazole resistance

A total of 3,756 isolates investigated in 53 studies were included in the analysis of cotrimoxazole resistance. The estimated average proportion based on the random-effects model was 0.034 (95% CI, 0.017 to 0.068), which differed significantly from zero (z = −8.955, *p* < 0.001). According to the Q-test, the actual outcomes appear to be heterogeneous (Q(52) = 520.558, I^2^ = 90.32%, *p* < 0.001).

**Fig 2:**
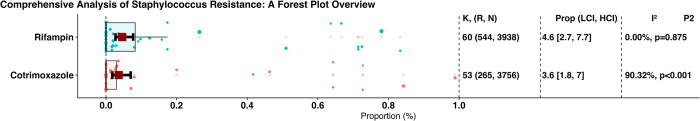
Overall worldwide antibiotic resistance proportion.

A forest plot showing the observed outcomes and the estimate based on the random-effects model is shown in Fig 2. With the fill-and-trim method implementation, the proportion changed to 0.064 (95% CI, 0.035 to 0.115). An examination of the studentized residuals revealed that none of the studies had a value larger than 3.307, indicating no outliers in the context of this model. However, Cook’s distances suggested that 1 study [31] could be considered overly influential.

**Fig 3:**
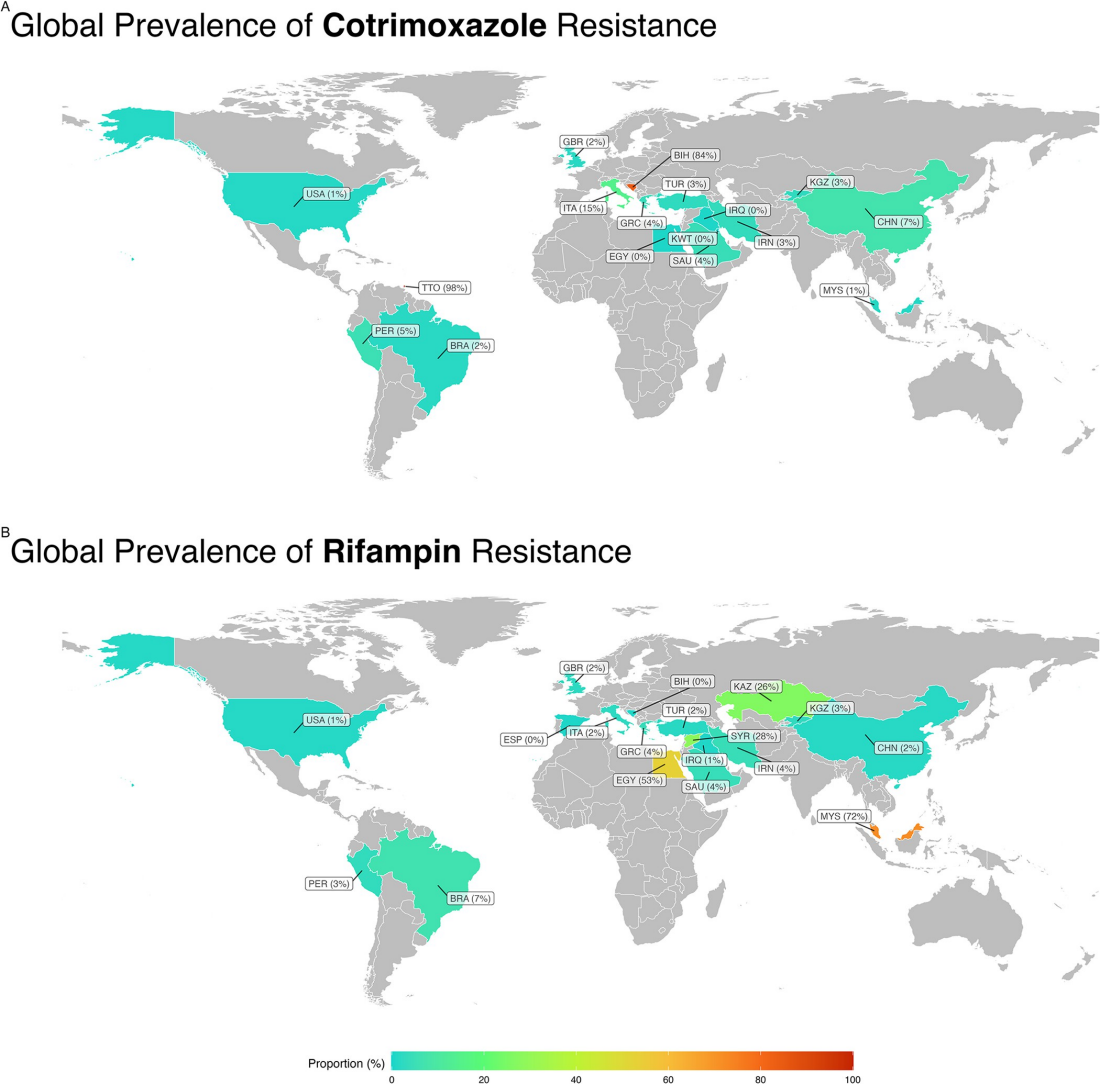
Funnel plot of each antibiotic meta-analysis (**A**) cotrimoxazole and (**B**) rifampin.

Both the rank correlation and the regression test indicated potential funnel plot asymmetry (*p* = 0.011 and *p* < 0.001, respectively) (Table 1 and Fig 3). Additionally, the Doi plot available in Fig A in S1 File revealed significant publication bias (LFK index = 2.44).

**Table 1:** Evaluation of publication bias in meta-analysis.

Antibiotic	Egger test	Begg test	Fail and safe	Trim and fill
**Cotrimoxazole**	*p* < 0.001	*p* = 0.011	8,173	0.064 (0.035, 0.115)
**Rifampin**	*p* < 0.001	*p* = 0.002	10,122	0.075 (0.047, 0.118)

This table provides a comprehensive assessment of potential publication bias in the meta-analysis using a range of statistical techniques. Included are statistics generated from Egger’s method, Begg’s method, the fail-safe N (NFS), and the trim-and-fill method. These methods are applied to investigate the presence of bias and its impact on the meta-analysis results, ensuring the robustness and reliability of the findings.

#### 3.2.2. Prevalence of rifampin resistance

A total of 3,938 isolates investigated across 60 studies were included in the analysis of rifampin resistance. Using a random-effects model, the estimated average proportion of resistance was 0.046 (95% CI: 0.027, 0.077), significantly different from zero (z = −10.822, *p* < 0.001). The Q-test indicated significant heterogeneity among the actual outcomes (Q(59) = 763.724, I^**2**^ = 92.39%, *p* < 0.001). After applying the fill-and-trim method, the proportion of rifampin resistance was adjusted to 0.075 (95% CI: 0.047, 0.118). Analysis of the studentized residuals revealed no studies with values larger than 3.341, indicating no outliers in this model.

However, Cook’s distances suggested that several studies [8,32,49,55,72,76] might be overly influential. Postremoval of these potential outliers, the proportion remained at 0.075 (95% CI: 0.047, 0.118). The rank correlation (*p* = 0.013) and the regression test (*p* = 0.012) indicated potential funnel plot asymmetry. Additionally, the Doi plot, shown in Fig A in S1 File, revealed a significant publication bias (LFK index = 1.99).

### 3.3. Subgroup analysis

**Fig 4:**
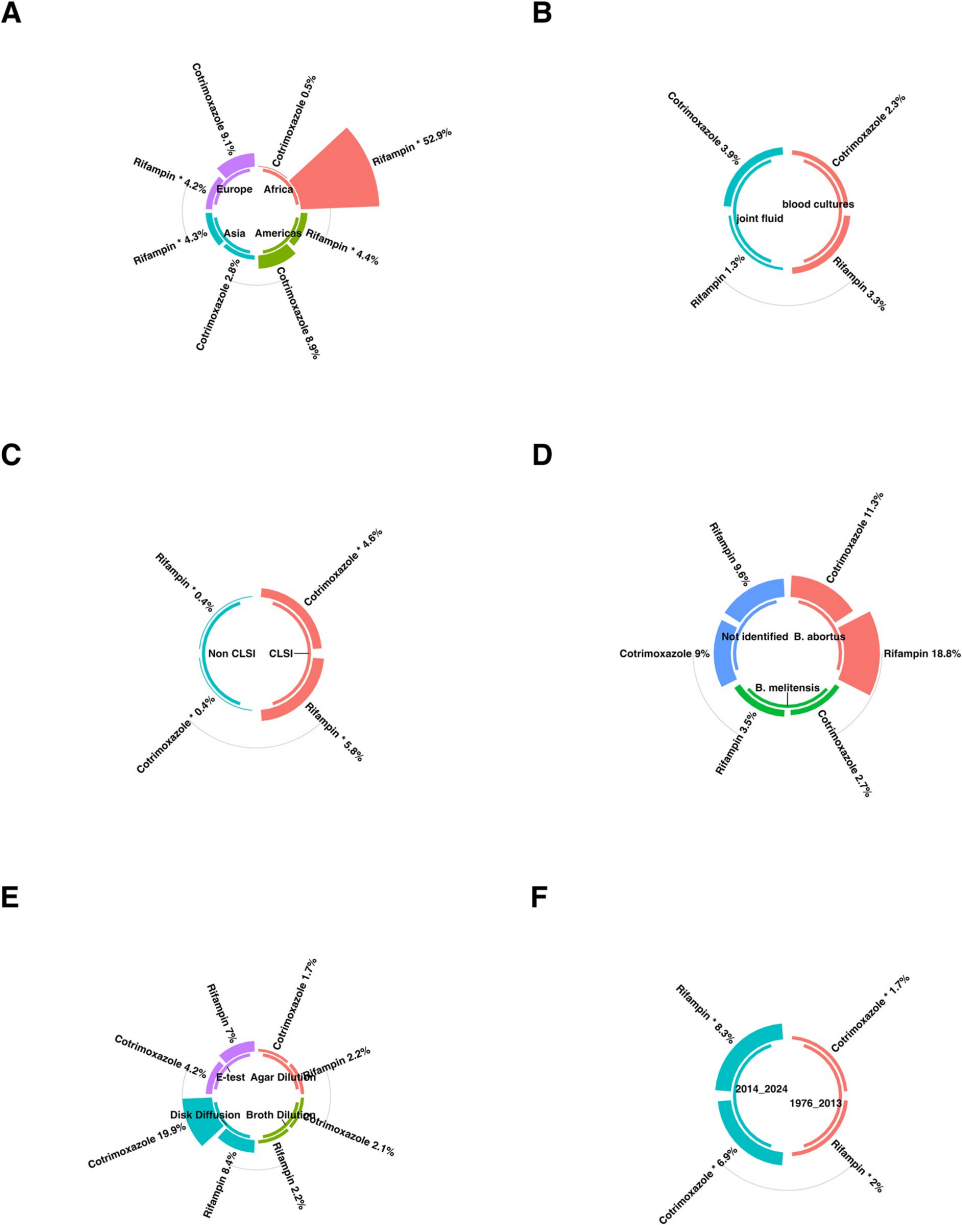
Subgroup analysis results were illustrated in figures. (**A**) Compression of the prevalence of antibiotic-resistant *Brucella* isolates between continents. (**B**) Compression of the prevalence of antibiotic-resistant *Brucella* isolates between specimens. (**C**) Compression of the prevalence of antibiotic-resistant *Brucella* isolates between AST guidelines. (**D**) Compression of the prevalence of antibiotic-resistant *Brucella* isolates between strains. (**E**) Compression of the prevalence of antibiotic-resistant *Brucella* isolates between AST methods. (**F**) Compression of the prevalence of *Brucella* isolates before and after 2020.

This passage provides an in-depth summary of subgroup analyses related to antibiotic resistance (Fig 4 and Table 2). It explores the variations in resistance rates across different regions, the impact of various AST methods, temporal trends, and the influence of study quality on reported findings. The subgroup analysis revealed that there was no statistically significant disparity in the prevalence of antibiotic resistance, including that of cotrimoxazole, rifampin, among various specimens (Fig 4B), strains (Fig 4D), and AST methods (Fig 4E).

**Table 2:** Prevalence of antibiotic resistance in the different subgroups.

Category	Subgroup	K (n, N)	Proportion 95%CI (LCI, HCI)	I^2^	P1	P2	P3
**Cotrimoxazole**							
**Overall**	NA	53 (265, 3,756)	0.034 (0.017, 0.068)	90.01%	*p* < 0.001	*p* < 0.001	NA
**Year group**	1976_2013	26 (97, 2,238)	0.016 (0.006, 0.044)	79.09%	*p* < 0.001	*p* < 0.001	*p* = 0.043
	2014_2024	27 (168, 1,518)	0.065 (0.027, 0.151)	91.74%	*p* < 0.001	*p* < 0.001	
**Countries**	Iraq	1 (0, 95)	0.005 (0.000, 0.078)	0.00%	*p* < 0.001	*p* > 0.999	*p* = 0.023
	Saudi Arabia	13 (35, 1,005)	0.043 (0.009, 0.186)	89.73%	*p* < 0.001	*p* < 0.001	
	Turkey	7 (4, 344)	0.022 (0.010, 0.047)	0.00%	*p* < 0.001	*p* = 0.921	
	Kuwait	2 (0, 480)	0.002 (0.000, 0.015)	0.00%	*p* < 0.001	*p* = 0.970	
	Greece	2 (2, 74)	0.033 (0.010, 0.109)	0.00%	*p* < 0.001	*p* = 0.881	
	Italy	2 (5, 32)	0.145 (0.006, 0.818)	79.03%	*p* = 0.288	*p* = 0.029	
	Iran	8 (6, 377)	0.034 (0.018, 0.065)	0.00%	*p* < 0.001	*p* = 0.437	
	China	4 (12, 202)	0.065 (0.021, 0.182)	65.03%	*p* < 0.001	*p* = 0.035	
	United States	1 (0, 39)	0.013 (0.001, 0.171)	0.00%	*p* = 0.002	*p* > 0.999	
	Peru	3 (19, 137)	0.052 (0.002, 0.656)	88.69%	*p* = 0.109	*p* < 0.001	
	Trinidad and Tobago	1 (87, 88)	0.989 (0.924, 0.998)	0.00%	*p* < 0.001	*p* > 0.999	
	Egypt	2 (0, 382)	0.005 (0.000, 0.058)	38.34%	*p* < 0.001	*p* = 0.203	
	Kyrgyzstan	1 (0, 17)	0.028 (0.002, 0.322)	0.00%	*p* = 0.013	*p* > 0.999	
	Malaysia	1 (0, 40)	0.012 (0.001, 0.167)	0.00%	*p* = 0.002	*p* > 0.999	
	Brazil	2 (2, 166)	0.015 (0.004, 0.052)	0.00%	*p* < 0.001	*p* = 0.698	
	United Kingdom	1 (2, 147)	0.014 (0.003, 0.053)	0.00%	*p* < 0.001	*p* > 0.999	
	Norway	1 (0, 23)	0.021 (0.001, 0.259)	0.00%	*p* = 0.007	*p* > 0.999	
	Bosnia and Herzegovina	1 (91, 108)	0.843 (0.761, 0.900)	0.00%	*p* < 0.001	*p* > 0.999	
**Continents**	Asia	37 (57, 2,560)	0.026 (0.013, 0.054)	82.24%	*p* < 0.001	*p* < 0.001	*p* = 0.282
	Europe	7 (100, 384)	0.086 (0.009, 0.487)	94.91%	*p* = 0.045	*p* < 0.001	
	Americas	7 (108, 430)	0.088 (0.009, 0.523)	92.49%	*p* = 0.059	*p* < 0.001	
	Africa	2 (0, 382)	0.005 (0.000, 0.058)	38.34%	*p* < 0.001	*p* = 0.203	
**Species**	*B*. *melitensis*	43 (151, 2,901)	0.026 (0.011, 0.056)	89.31%	*p* < 0.001	*p* < 0.001	*p* = 0.324
	Not identified	5 (92, 514)	0.087 (0.004, 0.686)	93.29%	*p* = 0.141	*p* < 0.001	
	*B*. *abortus*	5 (22, 341)	0.106 (0.011, 0.571)	93.41%	*p* = 0.084	*p* < 0.001	
**Specimens**	blood cultures	28 (114, 2,022)	0.022 (0.008, 0.062)	90.40%	*p* < 0.001	*p* < 0.001	*p* = 0.941
	joint fluid	1 (1, 37)	0.027 (0.004, 0.168)	0.00%	*p* < 0.001	*p* > 0.999	
**AST**	Agar Dilution	7 (6, 760)	0.014 (0.007, 0.027)	0.00%	*p* < 0.001	*p* = 0.671	*p* = 0.164
	E-test	31 (57, 1,937)	0.039 (0.018, 0.083)	83.30%	*p* < 0.001	*p* < 0.001	
	Broth Dilution	10 (96, 834)	0.021 (0.002, 0.168)	93.94%	*p* < 0.001	*p* < 0.001	
	Disk Diffusion	5 (106, 225)	0.205 (0.015, 0.815)	90.73%	*p* = 0.349	*p* < 0.001	
**Guidelines**	Non CLSI	6 (0, 699)	0.004 (0.001, 0.013)	0.00%	*p* < 0.001	*p* > 0.999	*p* = 0.046
	CLSI	47 (265, 3,057)	0.043 (0.021, 0.088)	90.26%	*p* < 0.001	*p* < 0.001	
**Rifampin**							
**Overall**	NA	60 (544, 3,938)	0.046 (0.027, 0.077)	92.39%	*p* < 0.001	*p* < 0.001	NA
**Year group**	1976_2013	27 (305, 1,976)	0.019 (0.006, 0.065)	93.62%	*p* < 0.001	*p* < 0.001	*p* = 0.039
	2014_2024	33 (239, 1,962)	0.077 (0.043, 0.133)	88.97%	*p* < 0.001	*p* < 0.001	
**Countries**	United Kingdom	2 (3, 168)	0.021 (0.007, 0.057)	0.00%	*p* < 0.001	*p* = 0.943	*p* = 0.036
	Iraq	2 (0, 151)	0.007 (0.001, 0.046)	0.00%	*p* < 0.001	*p* = 0.794	
	Saudi Arabia	11 (28, 817)	0.041 (0.007, 0.206)	89.40%	*p* < 0.001	*p* < 0.001	
	Spain	1 (0, 94)	0.005 (0.000, 0.079)	0.00%	*p* < 0.001	*p* > 0.999	
	Turkey	11 (2, 560)	0.014 (0.007, 0.030)	0.00%	*p* < 0.001	*p* = 0.984	
	Greece	2 (2, 74)	0.033 (0.010, 0.109)	0.00%	*p* < 0.001	*p* = 0.881	
	Italy	1 (0, 20)	0.024 (0.001, 0.287)	0.00%	*p* = 0.009	*p* > 0.999	
	Iran	9 (21, 378)	0.039 (0.007, 0.184)	85.21%	*p* < 0.001	*p* < 0.001	
	China	4 (1, 202)	0.013 (0.004, 0.043)	0.00%	*p* < 0.001	*p* = 0.974	
	United States	1 (0, 39)	0.013 (0.001, 0.171)	0.00%	*p* = 0.002	*p* > 0.999	
	Peru	3 (4, 137)	0.033 (0.006, 0.170)	53.38%	*p* < 0.001	*p* = 0.117	
	Syria	2 (62, 189)	0.280 (0.052, 0.734)	96.40%	*p* = 0.345	*p* < 0.001	
	Egypt	3 (291, 403)	0.529 (0.199, 0.836)	86.47%	*p* = 0.879	*p* < 0.001	
	Kyrgyzstan	1 (0, 17)	0.028 (0.002, 0.322)	0.00%	*p* = 0.013	*p* > 0.999	
	Malaysia	1 (29, 40)	0.725 (0.568, 0.841)	0.00%	*p* = 0.006	*p* > 0.999	
	Brazil	2 (6, 166)	0.058 (0.007, 0.347)	84.75%	*p* = 0.011	*p* = 0.010	
	Kazakhstan	1 (87, 329)	0.264 (0.220, 0.315)	0.00%	*p* < 0.001	*p* > 0.999	
	Norway	2 (8, 46)	0.174 (0.089, 0.311)	0.00%	*p* < 0.001	*p* > 0.999	
	Bosnia and Herzegovina	1 (0, 108)	0.005 (0.000, 0.069)	0.00%	*p* < 0.001	*p* > 0.999	
**Continents**	Europe	9 (13, 510)	0.039 (0.015, 0.095)	62.76%	*p* < 0.001	*p* = 0.006	*p* = 0.036
	Asia	42 (230, 2,683)	0.041 (0.022, 0.073)	88.14%	*p* < 0.001	*p* < 0.001	
	Americas	6 (10, 342)	0.041 (0.015, 0.108)	58.50%	*p* < 0.001	*p* = 0.034	
	Africa	3 (291, 403)	0.529 (0.199, 0.836)	86.47%	*p* = 0.879	*p* < 0.001	
**Species**	*B*. *melitensis*	51 (489, 3,366)	0.033 (0.018, 0.060)	92.14%	*p* < 0.001	*p* < 0.001	*p* = 0.102
	Not identified	4 (26, 231)	0.096 (0.008, 0.596)	91.93%	*p* = 0.095	*p* < 0.001	
	*B*. *abortus*	5 (29, 341)	0.180 (0.025, 0.648)	93.98%	*p* = 0.162	*p* < 0.001	
**Specimens**	blood cultures	30 (418, 2,264)	0.031 (0.014, 0.071)	93.92%	*p* < 0.001	*p* < 0.001	*p* = 0.707
	joint fluid	1 (0, 37)	0.013 (0.001, 0.178)	0.00%	*p* = 0.002	*p* > 0.999	
**AST**	Disk Diffusion	6 (20, 177)	0.079 (0.010, 0.411)	87.33%	*p* = 0.022	*p* < 0.001	*p* = 0.122
	Agar Dilution	6 (8, 652)	0.018 (0.010, 0.034)	0.00%	*p* < 0.001	*p* = 0.618	
	E-test	33 (449, 1,935)	0.066 (0.033, 0.128)	92.77%	*p* < 0.001	*p* < 0.001	
	Broth Dilution	15 (67, 1,174)	0.021 (0.006, 0.065)	89.13%	*p* < 0.001	*p* < 0.001	
**Guidelines**	CLSI	54 (544, 3,239)	0.054 (0.032, 0.092)	92.36%	*p* < 0.001	*p* < 0.001	*p* = 0.006
	Non CLSI	6 (0, 699)	0.004 (0.001, 0.013)	0.00%	*p* < 0.001	*p* > 0.999	

#### 3.3.1. Subgroup analysis based on continents

The subgroup analysis revealed a statistically significant disparity in the prevalence of antibiotic resistance, including that of rifampin, among various continents. The continent with the lowest resistance rate for antibiotic rifampin was Europe, exhibiting a prevalence rate of 3.9%. Conversely, the continent with the highest resistance rate was observed in Africa, with a prevalence rate reaching 52.9% (Fig 4A).

#### 3.3.2. Subgroup analysis based on AST guidelines

The subgroup analysis revealed a statistically significant disparity in antibiotic resistance prevalence, including cotrimoxazole and rifampin, among various guidelines. For the antibiotic cotrimoxazole, the guidelines with the lowest resistance rate were non-CLSI, exhibiting a prevalence rate of 0.4%. Conversely, the guidelines with the highest resistance rate were observed in CLSI, with a prevalence rate reaching 4.3%.

For the antibiotic rifampin, the guidelines with the lowest resistance rate were non-CLSI, exhibiting a prevalence rate of 0.4%. Conversely, the guidelines with the highest resistance rate were observed in CLSI, with a prevalence rate reaching 5.4% (Fig 4C).

#### 3.3.3. Subgroup analysis based on year group

The subgroup analysis revealed a statistically significant disparity in antibiotic resistance prevalence, including cotrimoxazole and rifampin, among various year groups. For the antibiotic cotrimoxazole, the year group with the lowest resistance rate was 1976 to 2013, exhibiting a prevalence rate of 1.6%. Conversely, the year group with the highest resistance rate was observed in 2014 to 2024, with a prevalence rate reaching 6.5%. For the antibiotic rifampin, the year group with the lowest resistance rate was 1976 to 2013, exhibiting a prevalence rate of 1.9%. Conversely, the year group with the highest resistance rate was observed in 2014 to 2024, with a prevalence rate reaching 7.7% (Fig 4F).

#### 3.3.4. Subgroup analysis based on countries

**Fig 5:**
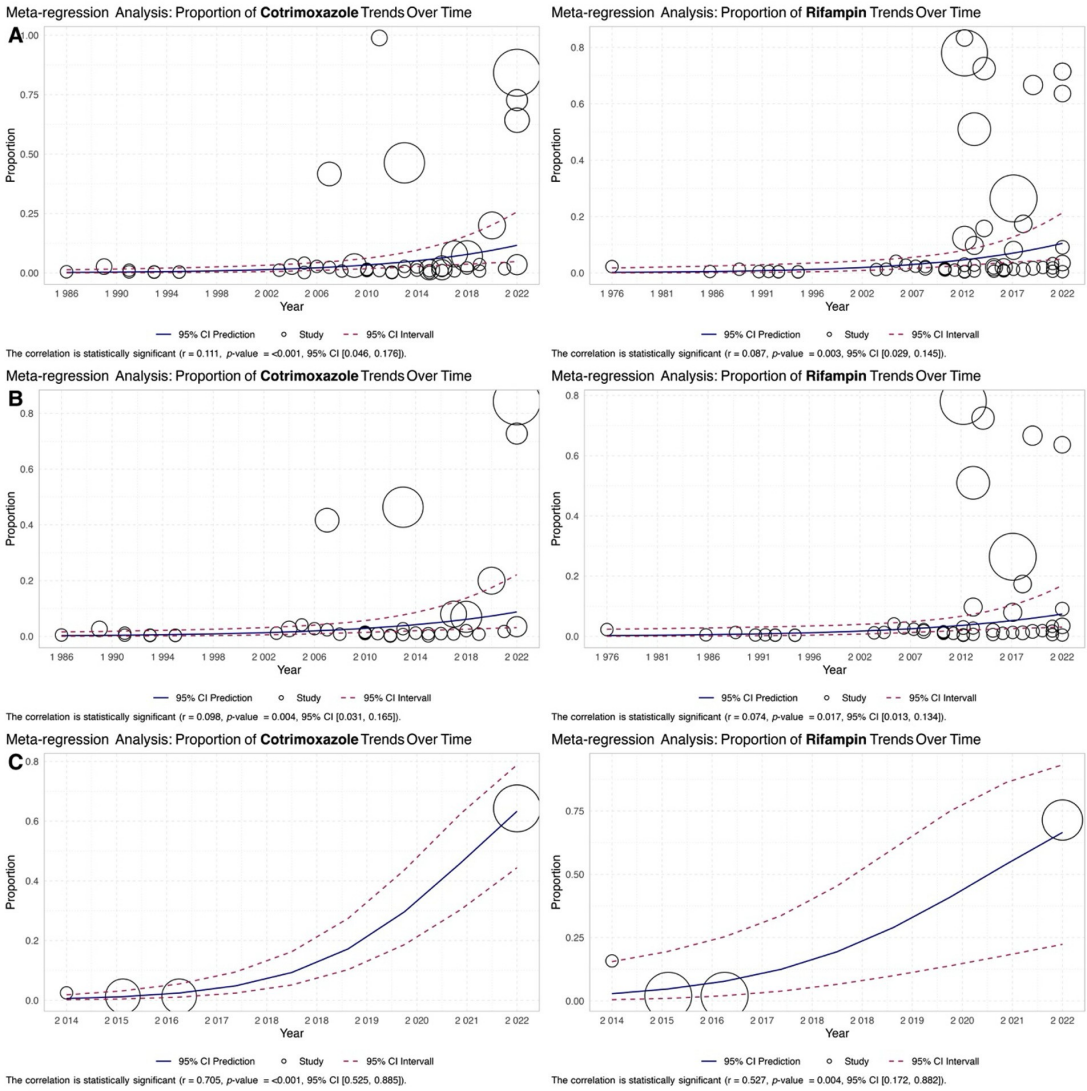
Worldwide map for prevalence of (**A**) cotrimoxazole and (**B**) rifampin. Global map visualization was created using OpenStreetMap data, available under the Open Database License (ODbL). Map data OpenStreetMap contributors, licensed under ODbL.

The subgroup analysis revealed a statistically significant disparity in antibiotic resistance prevalence, including cotrimoxazole and rifampin, among various countries. For the antibiotic cotrimoxazole, the country with the lowest rate of resistance was Kuwait, exhibiting a prevalence rate of 0.2%, while conversely, the country with the highest resistance rate was observed in Trinidad and Tobago, with a prevalence rate reaching 98.9% (Fig 5). For the antibiotic rifampin, Spain had the lowest rate of resistance, exhibiting a prevalence rate of 0.5%. Conversely, Malaysia had the highest resistance rate, reaching 72.5%.

### 3.4. Meta-regression analysis: Delving into the factors shaping antibiotic resistance in *Brucella* over time

**Fig 6:**
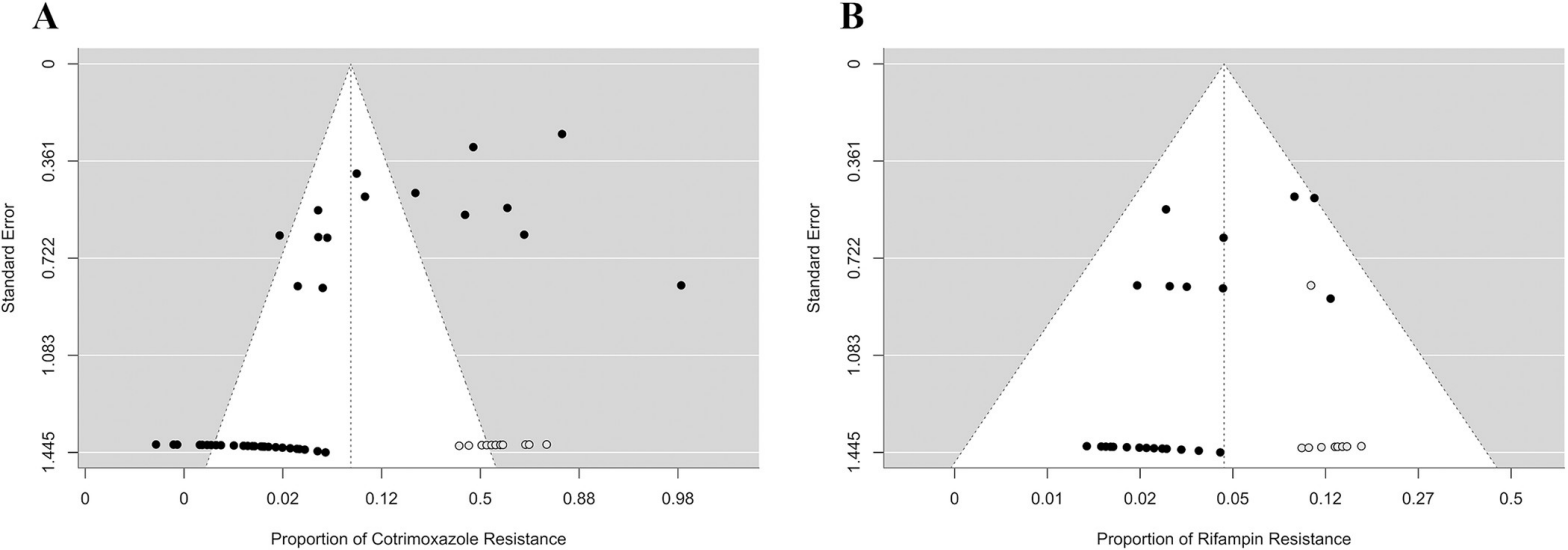
The meta-regression analysis results were illustrated in the scatter plot of the trend of the proportion of (**A**) cotrimoxazole and (**B**) rifampin resistance isolates over time.

The meta-regression results showed that the overall trend of the proportion of cotrimoxazole-resistant *Brucella* isolates increased over time (r = 0.111, *p* < 0.001), with a similar trend observed for rifampin resistance (r = 0.077, *p* = 0.008) (Fig 6).

## 4. Discussion

The comprehensive analysis spans 59 studies from 21 countries, capturing evidence on resistance patterns in *Brucella* isolates from 1976 to 2024. Our finding reveals the challenges of cotrimoxazole and rifampin resistance in pediatric brucellosis caused by *B*. *melitensis* and *B*. *abortus*. The determined average cotrimoxazole resistance proportion of 0.034 (95% CI, 0.017 to 0.068) highlights a noteworthy level of resistance, accompanied by significant heterogeneity (I^2^ = 90.01%), indicating diverse resistance patterns across studies. Adjusting for potential publication bias using the fill-and-trim method increased the proportion to 0.064. Meta-regression analysis reveals a significant temporal rise in cotrimoxazole (*p* = 0.001) and rifampin (*p* = 0.008) resistance, particularly notable in *B*. *melitensis* compared to *B*. *abortus*. This observed resistance prevalence raises concerns for *Brucella* and other bacterial infections, emphasizing the importance of continuous surveillance and prudent antibiotic utilization. The global scope of this concern is evident in sub-Saharan Africa, where studies report elevated levels of nonsusceptibility, underscoring the necessity for ongoing research and surveillance to address this critical public health issue effectively [83]. In addition, other studies, aligned with the current study, revealed the increasing resistance rates of these antibiotics over time [30,84]. Several factors could contribute to this trend, particularly the misuse and overuse of antibiotics in recent years. This misuse includes inappropriate prescribing practices, such as the use of antibiotics for viral infections, and the lack of adherence to treatment guidelines by both healthcare providers and patients. Additionally, the use of antibiotics in agriculture and animal husbandry has further accelerated the development of resistance.

Our findings align with broader global trends in antibiotic resistance observed in other pathogens. Similar increases in resistance rates over time have been documented in studies of rifampin and cotrimoxazole. These trends highlight the significant challenge posed by antibiotic resistance on a global scale, necessitating coordinated efforts in antimicrobial stewardship, education, and the development of new therapeutic strategies.

Similarly, a study in Ghana reported a very high prevalence of cotrimoxazole resistance, indicating the widespread nature of this issue [85]. The analysis of rifampin resistance, involving 3,938 isolates from 60 reports, showed an estimated average proportion of 0.046 (95% CI, 0.027, 0.077). High heterogeneity (I^2^ = 92.39%) suggests diverse rifampin resistance patterns, with the fill-and-trim method adjusting the proportion to 0.075, highlighting potential publication bias. Meta-regression analysis revealed a significant temporal increase in rifampin resistance, particularly pronounced in *B*. *melitensis* compared to *B*. *abortus*.

Concerns about rifampin resistance in *Brucella* are supported by evidence showing an increasing trend, especially in *B*. *melitensis* [30]. The high heterogeneity in resistance patterns underscores the need for further research to understand the mechanisms behind this trend [86]. Identifying mutations associated with antimicrobial resistance in Egypt emphasizes the molecular basis of resistance and the necessity for continuous surveillance [55]. The potential publication bias indicated by the fill-and-trim method underscores the importance of critically evaluating existing literature on rifampin resistance in *Brucella* [30]. The prevalence of rifampin resistance in *Brucella*, particularly in *B*. *melitensis*, is a growing concern supported by evidence from various regions [87]. This concern parallels the observed escalation in resistance rates over the years, emphasizing the urgent need for heightened control measures, systematic screening of *Brucella* treatment options, and monitoring antibiotic resistance trends in *Brucella* infections [88]. A comprehensive systematic review and meta-analysis by Shahrabi and colleagues [89] explored tetracycline resistance in *B*. *melitensis* and *B*. *abortus* across 51 studies from 1983 to 2020. The study reported a significant increase in resistance to tetracycline and doxycycline over time. This observed trend aligns with our present cotrimoxazole and rifampin resistance study, revealing a worrisome escalation in resistance rates over the years. The findings highlight the pressing need for heightened control measures, systematic screening of *Brucella* treatment options, and ongoing monitoring of antibiotic resistance trends in *Brucella* infections.

Meta-regression analysis was used to explore further the factors influencing antibiotic resistance trends. Over time, the increase of cotrimoxazole- and rifampin-resistant *Brucella* isolates emphasizes the need for continuous surveillance and intervention strategies. The species-specific trends revealed a higher increase in resistance for *B*. *melitensis* than for *B*. *abortus*, suggesting the necessity for targeted interventions against this species. Subgroup analysis offers valuable insights into the nuanced variations in antibiotic resistance prevalence based on diverse factors, providing a more granular understanding of the challenges posed by cotrimoxazole and rifampin resistance in *Brucella* isolates. The observed temporal variation in cotrimoxazole resistance is particularly striking, with the lowest recorded incidence from 1976 to 2013 (1.6%), contrasting sharply with the highest incidence in 2014 to 2024 (6.5%). This temporal escalation raises concerns regarding the recent surge in resistance, suggesting a potential shift in resistance dynamics over the past few years. In addition, for the antibiotic rifampin, the year group with the lowest resistance rate was 1976 to 2013, exhibiting a prevalence rate of 1.9%.

Conversely, the year group with the highest resistance rate was observed in 2014 to 2024, with a prevalence rate reaching 7.7%. The reasons for this temporal pattern warrant further exploration, and the findings underscore the importance of continuous surveillance in tracking and responding to evolving resistance trends.

Geographical differences in cotrimoxazole resistance are evident, with Kuwait displaying the lowest incidence at 0.2%, whereas Trinidad and Tobago reported the highest incidence at 98.9%. For the antibiotic rifampin, Spain had the lowest resistance rate, exhibiting a prevalence rate of 0.5%. Conversely, Malaysia had the highest resistance rate, reaching 72.5%.

These disparities highlight the impact of regional factors, including healthcare practices, antimicrobial use, and local epidemiological conditions, on resistance patterns. Understanding these geographical variations is crucial for tailoring interventions to specific regions and emphasizing the need for targeted surveillance and intervention strategies in high-resistance areas.

Continental analysis revealed substantial disparities in rifampin resistance, with Europe reporting the lowest incidence at 1.3%, whereas Africa demonstrated the highest recorded incidence at 52.9%. Diverse healthcare infrastructures, socioeconomic factors, and variations in antibiotic prescription practices may influence these continental differences. The higher incidence in Africa may also reflect challenges in healthcare access, diagnostic capabilities, and widespread use of antibiotics, emphasizing the importance of context-specific approaches to address resistance. Subgroup analysis based on the AST guidelines sheds light on the substantial differences in rifampin resistance. The non-CLSI guidelines exhibited the lowest recorded incidence at 0.4%, while the CLSI guidelines showed the highest incidence at 4.3%. For the antibiotic rifampin, the guidelines with the lowest resistance rate were non-CLSI, exhibiting a prevalence rate of 0.4%.

Conversely, the guidelines with the highest resistance rate were observed in CLSI, with a prevalence rate reaching 5.4%. These variations may arise from differences in laboratory methodologies, interpretative criteria, and local resistance patterns considered by the different guidelines. These findings underscore the importance of standardizing guidelines and highlight the need for harmonization to ensure consistency in resistance reporting and interpretation.

The subgroup analysis reinforces the complex nature of antibiotic resistance dynamics in *Brucella* isolates, influenced by temporal, geographical, and guideline-related factors. The observed disparities underscore the importance of tailoring interventions based on regional and temporal trends, emphasizing the need for targeted surveillance, antimicrobial stewardship programs, and global collaboration to address the multifaceted challenge of antibiotic resistance in brucellosis.

Due to the chronic nature of *Brucella* infection, extended treatment periods are required, heightening the risk of antibiotic resistance—consequently, all treatment guidelines for brucellosis advocate for combination therapy to inhibit resistance development [90]. Our study indicates a significant prevalence of resistance to cotrimoxazole and rifampin. Therefore, it is strongly recommended that antibiotic resistance in *Brucella* be routinely screened during both treatment and follow-up phases to address and manage this resistance issue effectively.

## 5. Conclusions

Our systematic review and meta-analysis revealed a concerning rise in cotrimoxazole and rifampin resistance in *B*. *melitensis* and *B*. *abortus*, posing a significant challenge to pediatric brucellosis management. Despite their historical efficacy, WHO-recommended first-line treatments face emerging resistance, particularly in Africa. The temporal, geographical, and species-specific variations underscore the dynamic nature of antibiotic resistance in *Brucella*, necessitating ongoing research, surveillance, and the development of targeted interventions to preserve the efficacy of these critical antibiotics in treating brucellosis. The comprehensive analysis, spanning 59 studies from 21 countries, highlights dynamic resistance patterns influenced by temporal, geographical, and guideline-related factors. Urgent measures are needed to address the escalating prevalence, considering local disparities and the necessity for standardized guidelines. Future research should focus on elucidating the specific factors contributing to these variations, such as the molecular mechanisms of resistance and the impact of treatment guidelines on resistance trends. This targeted approach will enable the development of effective strategies to curb the rise of resistance and ensure the continued efficacy of essential antibiotics in treating brucellosis.

## Supporting information

S1 File The Joanna Briggs Institute (JBI) critical appraisal checklist for analytical cross-sectional studies. (DOCX)
