# Supplementary material for: Unravelling the challenge of cotrimoxazole and rifampin resistance in B. melitensis and B. abortus: A systematic review and meta-analysis
Source: PLoS Negl Trop Dis. 2024 Dec 2;18(12):e0012630. doi: 10.1371/journal.pntd.0012630 (PMC11658705; doi:10.1371/journal.pntd.0012630)
Supplement: S1 File — (DOCX) [file pntd.0012630.s001.docx]

# Questionnaire A:

# The Joanna Briggs Institute (JBI) Critical Appraisal Checklist for Analytical Cross-Sectional Studies

|  | Yes | No | Unclear | Not applicable |
| --- | --- | --- | --- | --- |
| 1. Were the criteria for inclusion in the sample clearly defined? | □ | □ | □ | □ |
| 1. Were the study subjects and the setting described in detail? | □ | □ | □ | □ |
| 1. Was the exposure measured in a valid and reliable way? | □ | □ | □ | □ |
| 1. Were objective, standard criteria used for measurement of the condition? | □ | □ | □ | □ |
| 1. Were confounding factors identified? | □ | □ | □ | □ |
| 1. Were strategies to deal with confounding factors stated? | □ | □ | □ | □ |
| 1. Were the outcomes measured in a valid and reliable way? | □ | □ | □ | □ |
| 1. Was appropriate statistical analysis used? | □ | □ | □ | □ |

**Table A:**

| Author | Year | Yesear group | Country | Species | Specimens | AST | Guidelines | Total | Cotrimoxazole | Rifampin | Criteria.for.inclusion | Subjects.and.setting | Exposure.measured | Criteria.used.for.measurement | Confounding.factors | Strategies.to.deal.with.confound | Outcomes.measured | Statistical.analysis | Quality_group |
| --- | --- | --- | --- | --- | --- | --- | --- | --- | --- | --- | --- | --- | --- | --- | --- | --- | --- | --- | --- |
| Corbel, M. J.(1) | 1976 | 1976-2019 | UK | B.M | Other | D.D | CLSI | 21 |  | 0 | Yes | Yes | Yes | Yes | Unclear | Unclear | Yes | Yes | High Quality |
| Bosch, J., et al.(2) | 1986 | 1976-2019 | Iraq | B.M | BC | AD | Non CLSI | 95 | 0 | 0 | Yes | Yes | Yes | Yes | Yes | Yes | Yes | Yes | High Quality |
| Qadri, S. M., et al.(3) | 1989 | 1976-2019 | Saudi Arabia | B.M | BC | E-test | CLSI | 37 | 1 | 0 | Yes | Yes | Yes | Yes | No | No | Yes | Yes | Medium Quality |
| Qadri, S. M., et al.(4) | 1991 | 1976-2019 | Saudi Arabia | B.M | BC | AD | CLSI | 42 | 0 |  | Yes | No | No | Yes | Yes | Yes | Yes | No | Low Quality |
| Qadri, S. M. H., et al.(4) | 1991 | 1976-2019 | Saudi Arabia | B.M | Other | BD | Non CLSI | 105 | 0 | 0 | Yes | Yes | Yes | Yes | Yes | Yes | Yes | Yes | High Quality |
| Loza, E., et al.(5) | 1992 | 1976-2019 | Spain | B.M | Other | BD | CLSI | 94 |  | 0 | Unclear | Unclear | Yes | Yes | Yes | Yes | Yes | Yes | High Quality |
| Qadri, S. M., et al.(6) | 1993 | 1976-2019 | Saudi Arabia | B.M | BC | AD | CLSI | 126 | 0 | 0 | Yes | Yes | Yes | Yes | No | Unclear | Yes | No | Low Quality |
| Qadri, S. M., et al.(6) | 1993 | 1976-2019 | Saudi Arabia | B.M | NA | BD | Non CLSI | 105 | 0 | 0 | Yes | Yes | Yes | Yes | Yes | Yes | Yes | Yes | High Quality |
| Qadri, S. M., et al.(6) | 1993 | 1976-2019 | Saudi Arabia | B.M | NA | AD | CLSI | 146 | 0 |  | Yes | Yes | Yes | Yes | Yes | Yes | Yes | Yes | High Quality |
| Qadri, S. M. H., et al.(7) | 1995 | 1976-2019 | Saudi Arabia | B.M | BC | BD | Non CLSI | 139 | 0 | 0 | No | Yes | Yes | Yes | No | No | Unclear | No | Low Quality |
| Qadri, S. M., et al.(7) | 1995 | 1976-2019 | Saudi Arabia | B.M | BC | BD | Non CLSI | 116 | 0 | 0 | Yes | Yes | Unclear | Yes | Unclear | Unclear | Yes | Yes | Medium Quality |
| Bodur, H., et al.(8) | 2003 | 1976-2019 | Turkey | B.M | NA | E-test | CLSI | 41 | 0 | 0 | Unclear | Yes | Yes | Yes | Unclear | Unclear | Yes | Yes | Medium Quality |
| Baykam, N., et al.(9) | 2004 | 1976-2019 | Turkey | B.M | joint fluid | E-test | CLSI | 37 | 1 | 0 | Unclear | Yes | Yes | Yes | Unclear | Unclear | Yes | Yes | Medium Quality |
| Kose, S., et al.(10) | 2005 | 1976-2019 | Turkey | B.M | BC | E-test | CLSI | 11 | 0 | 0 | Yes | Yes | Yes | Unclear | Unclear | Yes | No | No | Low Quality |
| Dimitrov, T. S., et al.(11) | 2005 | 1976-2019 | Kuwait | ND | BC | E-test | CLSI | 249 | 0 |  | Yes | Unclear | Yes | Yes | Yes | Yes | Yes | Yes | High Quality |
| Turkmani, A., et al.(12) | 2006 | 1976-2019 | Greece | B.M | Other | E-test | CLSI | 17 | 0 | 0 | Yes | Yes | Yes | Yes | Yes | Unclear | Yes | Yes | High Quality |
| Cinzia Marianelli, et al.(13) | 2007 | 1976-2019 | Italy | B.M | BC | E-test | CLSI | 12 | 5 |  | Yes | Yes | Yes | Yes | No | Yes | Yes | Yes | High Quality |
| Marianelli, C., et al.(13) | 2007 | 1976-2019 | Italy | B.M | BC | E-test | CLSI | 20 | 0 | 0 | Yes | Yes | Yes | Yes | Yes | Yes | Yes | Yes | High Quality |
| Ayaşlioǧlu, E., et al.(14) | 2008 | 1976-2019 | Iran | B.M | BC | D.D | CLSI | 60 | 0 | 1 | Yes | Yes | Yes | Unclear | Unclear | Unclear | Unclear | Unclear | Low Quality |
| Sayan, M., et al.(15) | 2008 | 1976-2019 | Turkey | B.M | BC | E-test | CLSI | 21 |  | 0 | Yes | Yes | Yes | Yes | Yes | Yes | Yes | Yes | High Quality |
| Altun, B., et al.(16) | 2009 | 1976-2019 | Turkey | ND | NA | E-test | CLSI | 96 | 3 |  | Yes | Yes | Yes | Yes | Yes | Yes | Yes | Yes | High Quality |
| Jiang, H., et al.(17) | 2010 | 1976-2019 | China | B.M | BC | BD | CLSI | 31 | 0 | 0 | Yes | Yes | Yes | Yes | No | Unclear | Yes | No | Low Quality |
| Lonsway, D. R., et al. (18) | 2010 | 1976-2019 | USA | B.M | BC | BD | CLSI | 39 | 0 | 0 | Yes | Yes | Yes | Yes | No | Yes | Yes | No | Medium Quality |
| Castillo, R., et al.(19) | 2010 | 1976-2019 | Peru | B.M | BC | E-test | CLSI | 48 | 0 | 0 | Yes | Yes | Yes | Yes | Unclear | Unclear | Yes | Yes | High Quality |
| Ozhak-Baysan, B., et al.(20) | 2010 | 1976-2019 | Turkey | B.M | BC | E-test | CLSI | 36 | 0 | 0 | Yes | Yes | Yes | Yes | Yes | Yes | Yes | Yes | High Quality |
| Adesiyun, A. A., et al.(21) | 2011 | 1976-2019 | Trinidad and Tobago | ND | NA | D.D | CLSI | 88 | 87 |  | Yes | Yes | Yes | Yes | No | Yes | Yes | Yes | High Quality |
| Maves, R. C., et al.(22) | 2011 | 1976-2019 | Peru | B.M | BC | E-test | CLSI | 48 | 0 | 0 | Yes | Yes | Yes | Yes | Yes | Yes | Yes | Yes | High Quality |
| Safi, M., et al.(23) | 2012 | 1976-2019 | Syrian Arab Republic | ND | Other | BD | CLSI | 89 |  | 11 | Unclear | Unclear | Yes | Yes | Unclear | Unclear | Yes | Yes | Medium Quality |
| Yesayli, GÜLer, et al.(24) | 2012 | 1976-2019 | Turkey | B.M | BC | BD | CLSI | 34 |  | 1 | Yes | Yes | Yes | Yes | No | Yes | Yes | No | Medium Quality |
| Sayan, M., et al.(25) | 2012 | 1976-2019 | Turkey | B.M | BC | E-test | CLSI | 94 |  | 0 | Yes | Unclear | Yes | Yes | Unclear | Unclear | Yes | Yes | Medium Quality |
| Qadri, S. M. Hussain, et al.(26) | 2012 | 1976-2019 | Saudi Arabia | B.M | NA | BD | Non CLSI | 139 | 0 | 0 | Unclear | Yes | Yes | Yes | Unclear | Yes | Yes | Yes | High Quality |
| Abdel-Maksoud, M., et al.(27) | 2012 | 1976-2019 | Egypt | B.M | BC | E-test | CLSI | 355 | 0 | ### | Yes | Yes | Yes | Yes | Yes | Yes | Yes | Yes | High Quality |
| Rahmani, Mohammad Reza, et al.(28) | 2012 | 1976-2019 | Iran | ND | BC | D.D | CLSI | 18 |  | 15 | Yes | Yes | Yes | Yes | Yes | Yes | Yes | Yes | High Quality |
| Parlak, M., et al.(29) | 2013 | 1976-2019 | Turkey | B.M | Other | E-test | CLSI | 73 | 0 | 0 | Yes | No | No | Yes | Yes | Yes | Yes | No | Low Quality |
| Al-Mariri, A., et al.(30) | 2013 | 1976-2019 | Syrian Arab Republic | B.M | Other | BD | CLSI | 100 |  | 51 | Yes | Unclear | Yes | Unclear | Unclear | Unclear | Yes | Yes | Medium Quality |
| Kasymbekov, J., et al.(31) | 2013 | 1976-2019 | Kyrgyzstan | B.M | BC | E-test | CLSI | 17 | 0 | 0 | Yes | Yes | Yes | Yes | Yes | Yes | Unclear | Yes | High Quality |
| Ilhan, Z., et al.(32) | 2013 | 1976-2019 | Peru | B.M | Other | D.D | CLSI | 41 | 19 | 4 | Yes | Yes | Yes | Yes | Yes | Yes | Yes | Yes | High Quality |
| Hashim, R., et al.(33) | 2014 | 1976-2019 | Malaysia | B.M | BC | E-test | CLSI | 40 | 0 | 29 | No | Yes | Yes | Yes | Yes | Yes | Yes | Yes | High Quality |
| Neto, AlbiN Magalhães, et al.(34) | 2014 | 1976-2019 | Brazil | B. A | NA | E-test | CLSI | 19 | 0 | 3 | Unclear | Yes | Yes | Yes | Unclear | Yes | Yes | Yes | High Quality |
| Barbosa Pauletti, R., et al.(35) | 2015 | 1976-2019 | Brazil | B. A | Other | AD | CLSI | 147 | 2 | 3 | Unclear | Yes | Yes | Yes | Unclear | Unclear | Yes | Unclear | Medium Quality |
| Deshmukh, A., et al.(36) | 2015 | 1976-2019 | Kuwait | B.M | NA | E-test | CLSI | 231 | 0 |  | Yes | Yes | Yes | Yes | Yes | Yes | No | No | Medium Quality |
| Denk, A., et al.(37) | 2015 | 1976-2019 | Turkey | B.M | BC | AD | CLSI | 80 |  | 0 | Unclear | Yes | Yes | Yes | Unclear | Unclear | Yes | Yes | Medium Quality |
| Etiz, P., et al.(38) | 2015 | 1976-2019 | Turkey | B.M | NA | E-test | CLSI | 50 | 0 | 1 | Yes | Yes | Unclear | Yes | Yes | Yes | Yes | Yes | High Quality |
| Irajian, G. R., et al.(39) | 2016 | 1976-2019 | Iran | ND | NA | E-test | CLSI | 68 | 2 | 0 | No | Yes | Yes | Yes | No | No | Unclear | No | Low Quality |
| Razzaghi, R., et al.(40) | 2016 | 1976-2019 | Iran | B.M | BC | E-test | CLSI | 48 | 0 | 0 | Yes | Yes | Yes | Yes | Yes | Yes | Yes | Yes | High Quality |
| Minharro, Silvia, et al.(41) | 2016 | 1976-2019 | United Kingdom | B. A | NA | AD | CLSI | 147 | 2 | 3 | Yes | Yes | Yes | Yes | Yes | Yes | Yes | Yes | High Quality |
| Torkaman Asadi, F., et al.(42) | 2017 | 1976-2019 | Iran | B.M | BC | E-test | CLSI | 55 | 0 |  | Yes | Yes | Yes | Yes | Yes | Unclear | Yes | Yes | High Quality |
| Torkaman Asadi, F., et al.(42) | 2017 | 1976-2019 | Iran | B.M | BC | E-test | CLSI | 37 |  | 0 | Yes | Yes | Yes | Yes | Yes | Yes | Yes | Yes | High Quality |
| Shevtsov, A., et al.(43) | 2017 | 1976-2019 | Kazakhstan | B.M | BC | E-test | CLSI | 329 |  | 87 | Yes | Yes | Yes | Yes | Yes | Yes | Yes | Yes | High Quality |
| Mohammadi Azad, Z., et al.(44) | 2017 | 1976-2019 | Iran | B.M | BC | E-test | CLSI | 50 | 4 | 4 | Yes | Yes | Yes | Yes | Yes | Yes | Yes | Yes | High Quality |
| Johansen, T. B., et al.(45) | 2018 | 1976-2019 | Nrway | B.M | NA | E-test | CLSI | 23 | 0 | 4 | No | Yes | Yes | Yes | No | No | Unclear | No | Low Quality |
| Liu, Z. G., et al.(46) | 2018 | 1976-2019 | China | B.M | BC | E-test | CLSI | 85 | 6 | 1 | Yes | Yes | Yes | Yes | No | Yes | Yes | No | Medium Quality |
| Johansen, T. B., et al.(45) | 2018 | 1976-2019 | Nrway | B.M | NA | BD | CLSI | 23 |  | 4 | Yes | Yes | Yes | Yes | Yes | Yes | Unclear | Yes | High Quality |
| Nb, et al.(47) | 2019 | 1976-2019 | Iran | ND | BC | D.D | CLSI | 13 | 0 |  | Unclear | Yes | Yes | Unclear | Unclear | Unclear | Yes | Yes | Medium Quality |
| Alamian, S., et al.(48) | 2019 | 1976-2019 | Iran | B.M | BC | E-test | CLSI | 60 | 0 | 1 | Unclear | Yes | Yes | Yes | Unclear | Unclear | Yes | Yes | Medium Quality |
| Khan, A. U., et al.(49) | 2019 | 1976-2019 | Egypt | B.M | NA | E-test | CLSI & EUCAST | 21 |  | 14 | Yes | Unclear | Yes | Yes | Yes | Yes | Yes | Yes | High Quality |
| Yesuan, H. T., et al.(50) | 2020 | 2020-2022 | China | B.M | BC | BD | CLSI | 25 | 5 | 0 | Yes | No | Yes | No | Yes | Yes | Yes | Yes | Medium Quality |
| Dadar, Maryam, et al.(51) | 2021 | 2020-2022 | Iran | B.M | NA | D.D | CLSI | 14 |  | 0 | Yes | Yes | Unclear | Unclear | Unclear | Unclear | Unclear | Yes | Low Quality |
| Gultekin, E., et al.(52) | 2021 | 2020-2022 | Turkey | B.M | NA | E-test | CLSI | 83 |  | 0 | Yes | Unclear | Yes | Yes | Yes | Yes | Yes | Yes | High Quality |
| Wareth, G., et al.(53) | 2021 | 2020-2022 | Egypt | B.M | Other | BD | CLSI | 27 | 0 | 0 | Yes | Yes | Yes | Yes | Yes | Yes | Yes | Yes | High Quality |
| Elbehiry, Ayman, et al.(54) | 2022 | 2020-2022 | Saudi Arabia | B. A | NA | E-test | CLSI | 14 | 9 | 10 | Unclear | Yes | Yes | Unclear | Unclear | No | No | No | Low Quality |
| Elbehiry, A., et al.(54) | 2022 | 2020-2022 | Saudi Arabia | B. A | Other | E-test | CLSI | 14 | 9 | 10 | Yes | Yes | Yes | Yes | No | No | Unclear | Unclear | Low Quality |
| Elbehiry, A., et al.(54) | 2022 | 2020-2022 | Saudi Arabia | B.M | Other | E-test | CLSI | 11 | 8 | 7 | Unclear | Yes | Yes | Yes | Unclear | Unclear | Yes | Yes | Medium Quality |
| Tscherne, A., et al.(55) | 2022 | 2020-2022 | Greece | B.M | BC | AD | CLSI & EUCAST | 57 | 2 | 2 | Yes | Yes | Yes | Yes | Yes | Yes | Yes | Yes | High Quality |
| Tscherne, A., et al.(55) | 2022 | 2020-2022 | Greece | B.M | BC | BD | CLSI & EUCAST | 57 | 2 | 2 | Yes | Yes | Yes | Yes | Yes | Yes | Yes | Yes | High Quality |
| Arapovic, J., et al.(56) | 2022 | 2020-2022 | Bosnia and Herzegovina | B.M | BC | BD | CLSI | 108 | 91 | 0 | Yes | Yes | Yes | Yes | Yes | Yes | Yes | Yes | High Quality |
| Elbehiry, Ayman, et al.(54) | 2022 | 2020-2022 | Saudi Arabia | B.M | NA | E-test | CLSI | 11 | 8 | 1 | Yes | Yes | Yes | Yes | Yes | Yes | Yes | Yes | High Quality |
| Maryam Dadar, et al. (57) | 2023 | 2014_2024 | Iran | B.M | NA | Disk Diffusion | CLSI | 23 | 0 | 0 | Yes | Yes | Yes | Yes | No | No | Yes | No | Low Quality |
| Han-Rui Ma, et al. (58) | 2023 | 2014_2024 | China | B.M | NA | E-test | CLSI | 61 | 1 | 0 | Yes | Yes | Some concern | Yes | No | No | Yes | Some concern | Low Quality |
| Khitam F Abbas, et al. (59) | 2024 | 2014_2024 | Iraq | ND | NA | E-test | CLSI | 56 |  | 0 | Yes | Yes | Yes | Yes | Yes | Yes | Yes | Yes | High Quality |

DD: Disk Diffusion, BC: Blood culture, BD: Broth Dilution, AD: Agar Dilution, ND: Not determined, B.M: *B. melitensis*, B.A: *B. abortus*.

**A** **B**


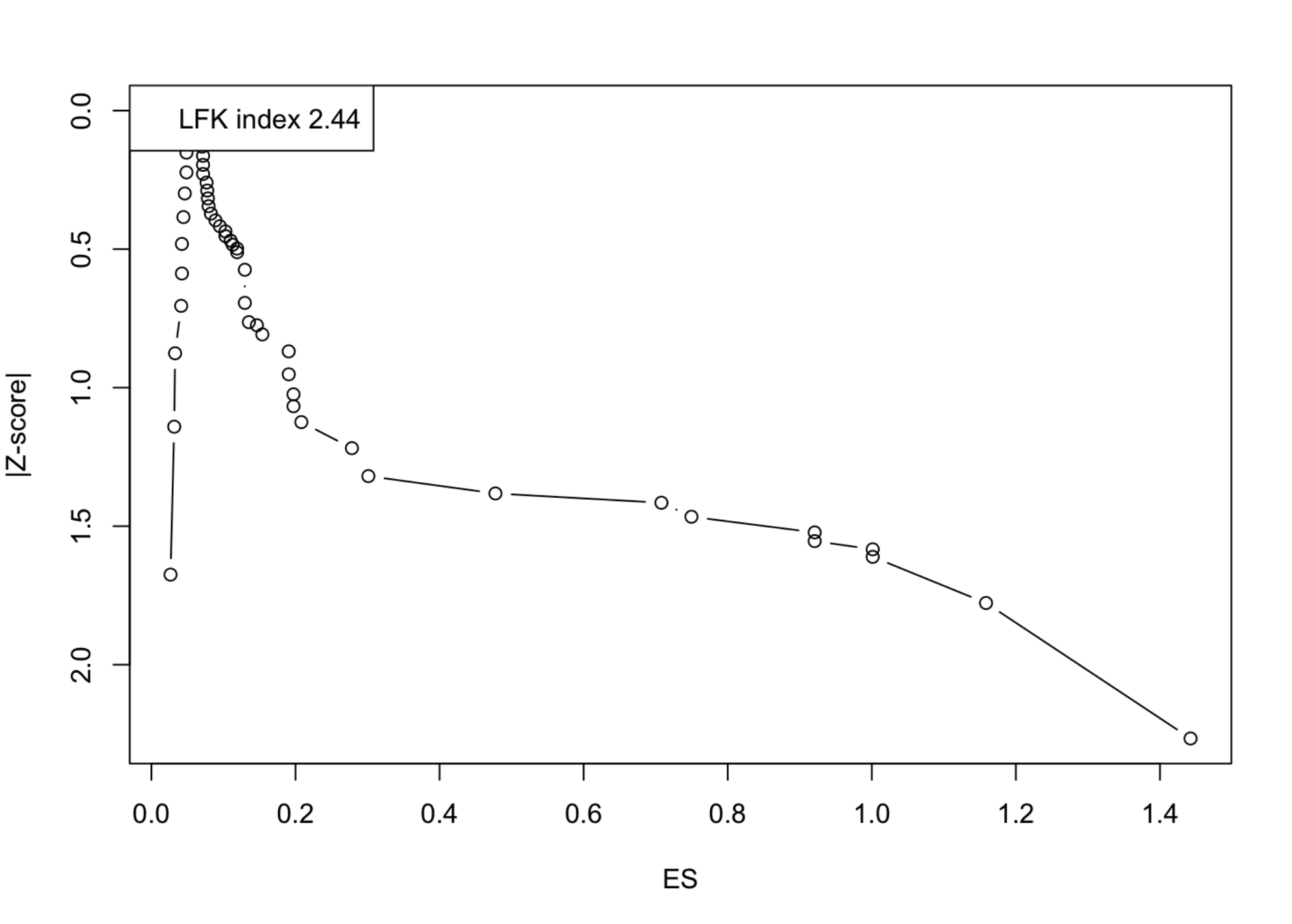

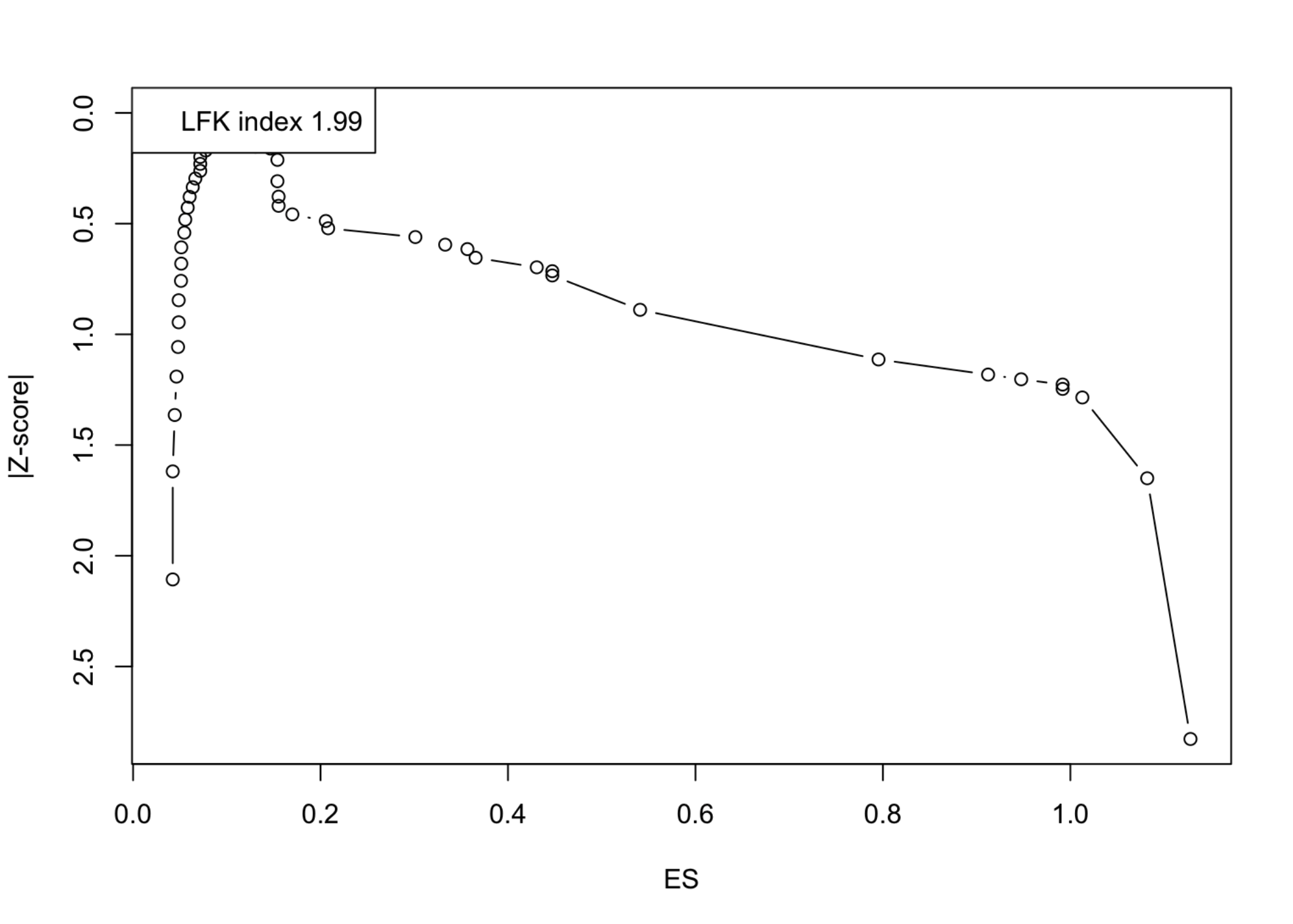


**Fig A:** The funnel plot for assessment of publication bias. **A:** Cotrimoxazole, **B:** Rifampin.

1. Corbel MJ. Determination of the in vitro sensitivity of Brucella strains to rifampicin. Br Vet J. 1976;132(3):266-75.

2. Bosch J, Linares J, Lopez de Goicoechea MJ, Ariza J, Cisnal MC, Martin R. In-vitro activity of ciprofloxacin, ceftriaxone and five other antimicrobial agents against 95 strains of Brucella melitensis. J Antimicrob Chemother. 1986;17(4):459-61.

3. Qadri SM, Akhtar M, Ueno Y, al-Sibai MB. Susceptibility of Brucella melitensis to fluoroquinolones. Drugs Exp Clin Res. 1989;15(10):483-5.

4. Qadri SM, Lee GC, Ellis ME. In vitro activity of lomefloxacin, a difluorinated quinolone, compared with other antimicrobials. Chemotherapy. 1991;37(3):166-74.

5. Loza E, Martínez Beltrán J, Baquero F, Leon A, Canton R, Garijo B, et al. Comparative in vitro activity of clarithromycin. European Journal of Clinical Microbiology and Infectious Diseases. 1992;11:856-66.

6. Qadri SM, Akhter J, Ueno Y, Saldin H. In vitro activity of eight fluoroquinolones against clinical isolates of Brucella melitensis. Ann Saudi Med. 1993;13(1):37-40.

7. Qadri SM, Halim MA, Ueno Y, Abumustafa FM, Postle AG. Antibacterial activity of azithromycin against Brucella melitensis. Chemotherapy. 1995;41(4):253-6.

8. Bodur H, Balaban N, Aksaray S, Yetener V, Akinci E, Colpan A, et al. Biotypes and antimicrobial susceptibilities of Brucella isolates. Scand J Infect Dis. 2003;35(5):337-8.

9. Baykam N, Esener H, Ergonul O, Eren S, Celikbas AK, Dokuzoguz B. In vitro antimicrobial susceptibility of Brucella species. Int J Antimicrob Agents. 2004;23(4):405-7.

10. Kose S, Kilic S, Ozbel Y. Identification of Brucella species isolated from proven brucellosis patients in Izmir, Turkey. J Basic Microbiol. 2005;45(4):323-7.

11. Dimitrov TS, Panigrahi D, Emara M, Al-Nakkas A, Awni F, Passadilla R. Incidence of bloodstream infections in a speciality hospital in Kuwait: 8-year experience. Med Princ Pract. 2005;14(6):417-21.

12. Turkmani A, Ioannidis A, Christidou A, Psaroulaki A, Loukaides F, Tselentis Y. In vitro susceptibilities of Brucella melitensis isolates to eleven antibiotics. Ann Clin Microbiol Antimicrob. 2006;5:1-4.

13. Marianelli C, Graziani C, Santangelo C, Xibilia MT, Imbriani A, Amato R, et al. Molecular epidemiological and antibiotic susceptibility characterization of Brucella isolates from humans in Sicily, Italy. J Clin Microbiol. 2007;45(9):2923-8.

14. Ayaşlioǧlu E, Kiliç S, Aydin K, Kiliç D, Kaygusuz S, Aǧalar C. Antimicrobial susceptibility of Brucella melitensis isolates from blood samples. Turk J Med Sci. 2008;38(3):257-62.

15. Sayan M, Yumuk Z, Dündar D, Bilenoğlu O, Erdenliğ S, Yaşvar E, et al. Rifampicin resistance phenotyping of Brucella melitensis by rpoB gene analysis in clinical isolates. Journal of chemotherapy. 2008;20(4):431-5.

16. Altun B, Hasçelik G, Gür D. In vitro activity of tigecycline against Brucella spp. Med J Trakya Univ. 2009;26(3):261-3.

17. Jiang H, Mao LL, Zhao HY, Li LY, Piao DR, Yao WQ, et al. MLVA typing and antibiotic susceptibility of Brucella human isolates from Liaoning, China. Trans R Soc Trop Med Hyg. 2010;104(12):796-800.

18. Lonsway DR, Jevitt LA, Uhl JR, Cockerill FR, 3rd, Anderson ME, Sullivan MM, et al. Effect of carbon dioxide on broth microdilution susceptibility testing of Brucella spp. J Clin Microbiol. 2010;48(3):952-6.

19. Castillo R, Guillen A, Espinosa B, Meza R, Espinoza N, Nunez G, et al. Antimicrobial Susceptibility of Brucella melitensis Isolates in Peru. ASM. 2010;110:A-023.

20. Ozhak-Baysan B, Ongut G, Ogunc D, Gunseren F, Sepin-Ozen N, Ozturk F, et al. Evaluation of in vitro activities of tigecycline and various antibiotics against Brucella spp. Pol J Microbiol. 2010;59(1):55-60.

21. Adesiyun AA, Baird K, Stewart-Johnson A. Antimicrobial resistance, phenotypic characteristics and phage types of B. abortus strains isolated from cattle and water buffalo (Bubalus bubalis) in Trinidad. Veterinarski Arhiv. 2011;81(3):391-404.

22. Maves RC, Castillo R, Guillen A, Espinosa B, Meza R, Espinoza N, et al. Antimicrobial susceptibility of Brucella melitensis isolates in Peru. Antimicrob Agents Chemother. 2011;55(3):1279-81.

23. Safi M, Al-Mariri A. Efficacy evaluation of some antibiotics against syrian brucella spp isolates, in vitro. Braz J Microbiol. 2012;43(4):1269-73.

24. Yayli G, AkÇAm FZ, Kaya O. Investigation of the in vitro activities of various antibiotics against Brucella melitensis strains. Turk J Med Sci. 2012;42(1):145-8.

25. Sayan M, Kilic S, Uyanik MH. Epidemiological survey of rifampicin resistance in clinic isolates of Brucella melitensis obtained from all regions of Turkey. J Infect Chemother. 2012;18(1):41-6.

26. Qadri SMH, Ueno Y. Susceptibility of Brucella melitensis to the New Fluoroquinolone CI-960. Drug Investig. 2012;3(5):365-7.

27. Abdel-Maksoud M, House B, Wasfy M, Abdel-Rahman B, Pimentel G, Roushdy G, et al. In vitro antibiotic susceptibility testing of Brucella isolates from Egypt between 1999 and 2007 and evidence of probable rifampin resistance. Ann Clin Microbiol Antimicrob. 2012;11:24.

28. Rahmani MR, Rashidi A, Hakhamaneshi MS, Shapouri R, Zandi F, Hazhir MS, et al. Evaluation of the antibacterial activity of Zataria multiflora Boiss., Rhus coriaria L. (sumac), Mentha piperita L., and Ocimum basilicum L. extracts on Brucella strains isolated from brucellosis patients. Turk J Med Sci. 2012;42(5):816-22.

29. Parlak M, Guducuoglu H, Bayram Y, Cikman A, Aypak C, Kilic S, et al. Identification and determination of antibiotic susceptibilities of Brucella strains isolated from patients in van, Turkey by conventional and molecular methods. Int J Med Sci. 2013;10(10):1406-11.

30. Al-Mariri A, Safi M. Effect of Medium pH on Antibiotic Activity against Syrian Brucella spp. Isolates. Iran J Med Sci. 2013;38(3):248-54.

31. Kasymbekov J, Imanseitov J, Ballif M, Schurch N, Paniga S, Pilo P, et al. Molecular epidemiology and antibiotic susceptibility of livestock Brucella melitensis isolates from Naryn Oblast, Kyrgyzstan. PLoS Negl Trop Dis. 2013;7(2):e2047.

32. Ilhan Z, Solmaz H, Ekin IH. In vitro antimicrobial susceptibility of Brucella melitensis isolates from sheep in an area endemic for human brucellosis in Turkey. J Vet Med Sci. 2013;75(8):1035-40.

33. Hashim R, Ahmad N, Mohamed Zahidi J, Tay BY, Mohd Noor A, Zainal S, et al. Identification and in vitro antimicrobial susceptibility of Brucella species isolated from human brucellosis. Int J Microbiol. 2014;2014:596245.

34. Neto AM, Corção G, Dasso MG, Keid LB, Costa Md. ANTIMICROBIAL SUSCEPTIBILITY PROFILE OF Brucella spp. ISOLATED IN BRAZIL. Revista de Patologia Tropical. 2014;43(2):163-72.

35. Barbosa Pauletti R, Reinato Stynen AP, Pinto da Silva Mol J, Seles Dorneles EM, Alves TM, de Sousa Moura Souto M, et al. Reduced Susceptibility to Rifampicin and Resistance to Multiple Antimicrobial Agents among Brucella abortus Isolates from Cattle in Brazil. PLoS One. 2015;10(7):e0132532.

36. Deshmukh A, Hagen F, Sharabasi OA, Abraham M, Wilson G, Doiphode S, et al. In vitro antimicrobial susceptibility testing of human Brucella melitensis isolates from Qatar between 2014 - 2015. BMC Microbiol. 2015;15:121.

37. Denk A, Demirdag K, Kalkan A, Ozden M, Cetinkaya B, Kilic SS. In vitro activity of Brucella melitensis isolates to various antimicrobials in Turkey. Infect Dis (Lond). 2015;47(6):364-9.

38. Etiz P, Kibar F, Ekenoglu Y, Yaman A. Characterization of antibiotic susceptibility of brucella spp isolates with E-Test method. Arch Clin Microbiol. 2015;6(1).

39. Irajian GR, Masjedian Jazi F, Mirnejad R, Piranfar V, Zahraei Salehi T, Amir Mozafari N, et al. Species-specific PCR for the Diagnosis and Determination of Antibiotic Susceptibilities of Brucella Strains Isolated from Tehran, Iran. Iran J Pathol. 2016;11(3):238-47.

40. Razzaghi R, Rastegar R, Momen-Heravi M, Erami M, Nazeri M. Antimicrobial susceptibility testing of Brucella melitensis isolated from patients with acute brucellosis in a centre of Iran. Indian J Med Microbiol. 2016;34(3):342-5.

41. Minharro S, Pauletti RB, Stynen APR, Mol JPDS, Dorneles EMS, Alves TM, et al. Minimal Inhibitory Concentration (MIC50, MIC90 and range) values of Brucella abortus strains isolated from cattle in Brazil, 1977-2009, to eight antimicrobials used in the treatment of human brucellosis. Figshare. 2016.

42. Torkaman Asadi F, Hashemi SH, Alikhani MY, Moghimbeigi A, Naseri Z. Clinical and Diagnostic Aspects of Brucellosis and Antimicrobial Susceptibility of Brucella Isolates in Hamedan, Iran. Jpn J Infect Dis. 2017;70(3):235-8.

43. Shevtsov A, Syzdykov M, Kuznetsov A, Shustov A, Shevtsova E, Berdimuratova K, et al. Antimicrobial susceptibility of Brucella melitensis in Kazakhstan. Antimicrob Resist Infect Control. 2017;6:130.

44. Mohammadi Azad Z, Moravej H, Fasihi-Ramandi M, Masjedian F, Nazari R, Mirnejad R, et al. In vitro synergistic effects of a short cationic peptide and clinically used antibiotics against drug-resistant isolates of Brucella melitensis. J Med Microbiol. 2017;66(7):919-26.

45. Johansen TB, Scheffer L, Jensen VK, Bohlin J, Feruglio SL. Whole-genome sequencing and antimicrobial resistance in Brucella melitensis from a Norwegian perspective. Sci Rep. 2018;8(1):8538.

46. Liu ZG, Di DD, Wang M, Liu RH, Zhao HY, Piao DR, et al. In vitro antimicrobial susceptibility testing of human Brucella melitensis isolates from Ulanqab of Inner Mongolia, China. BMC Infect Dis. 2018;18(1):43.

47. Nob, egani AS, Motamedifar M. ANTIBIOTIC SENSITIVITY PROFILE OF THE BACTERIAL ISOLATES FROM THE BLOOD SAMPLES OF THE PATIENTS IN DIFFERENT WARDS OF A MAJOR REFERRAL HOSPITAL, SHIRAZ, IRAN 2015-2016. Pharmacophore. 2019;10(2):30-6.

48. Alamian S, Dadar M, Etemadi A, Afshar D, Alamian MM. Antimicrobial susceptibility of Brucella spp. isolated from Iranian patients during 2016 to 2018. Iran J Microbiol. 2019;11(5):363-7.

49. Khan AU, Shell WS, Melzer F, Sayour AE, Ramadan ES, Elschner MC, et al. Identification, Genotyping and Antimicrobial Susceptibility Testing of Brucella spp. Isolated from Livestock in Egypt. Microorganisms. 2019;7(12).

50. Yuan HT, Wang CL, Liu LN, Wang D, Li D, Li ZJ, et al. Epidemiologically characteristics of human brucellosis and antimicrobial susceptibility pattern of Brucella melitensis in Hinggan League of the Inner Mongolia Autonomous Region, China. Infect Dis Poverty. 2020;9(1):79.

51. Dadar M, Bazrgari N, Garosi GA, Hassan S. Investigation of Mutations in the Rifampin-Resistance-Determining Region of the rpoB Gene of Brucella melitensis by Gene Analysis. JJM. 2021;14(2).

52. Gultekin E, Uyanik MH, Albayrak A, Kilic S. Investigation of antibiotic susceptibilities of Brucella Strains isolated from various clinical samples in eastern Turkey. Eur J Med Res. 2021;26(1):57.

53. Wareth G, El-Diasty M, Abdel-Hamid NH, Holzer K, Hamdy MER, Moustafa S, et al. Molecular characterization and antimicrobial susceptibility testing of clinical and non-clinical Brucella melitensis and Brucella abortus isolates from Egypt. One Health. 2021;13:100255.

54. Elbehiry A, Aldubaib M, Al Rugaie O, Marzouk E, Abaalkhail M, Moussa I, et al. Proteomics-based screening and antibiotic resistance assessment of clinical and sub-clinical Brucella species: An evolution of brucellosis infection control. PLoS One. 2022;17(1):e0262551.

55. Tscherne A, Mantel E, Boskani T, Budniak S, Elschner M, Fasanella A, et al. Adaptation of Brucella melitensis Antimicrobial Susceptibility Testing to the ISO 20776 Standard and Validation of the Method. Microorganisms. 2022;10(7).

56. Arapovic J, Kompes G, Dedic K, Teskeredzic S, Ostojic M, Travar M, et al. Antimicrobial resistance profiles of human Brucella melitensis isolates in three different microdilution broths: the first multicentre study in Bosnia and Herzegovina. J Glob Antimicrob Resist. 2022;29:99-104.

57. Dadar M, Tabibi R, Alamian S, Caraballo-Arias Y, Mrema EJ, Mlimbila J, et al. Safety concerns and potential hazards of occupational brucellosis in developing countries: a review. Journal of Public Health. 2023;31(10):1681-90.

58. Ma H-r, Xu H-j, Wang X, Bu Z-y, Yao T, Zheng Z-r, et al. Molecular characterization and antimicrobial susceptibility of human Brucella in Northeast China. Frontiers in Microbiology. 2023;14:1137932.

59. Abbas KF, Abdul-Hassan LS, Hadi OM, Almulla AF, Kathair ZY. Study of some cytokines in patients with brucellosis in Najaf province. Anaesthesia, Pain & Intensive Care. 2024;28(1):39-43.
